# Assessment of right ventricular structure and systolic function in amateur marathon runners using three-dimensional speckle tracking echocardiography

**DOI:** 10.1007/s10554-023-02869-z

**Published:** 2023-05-13

**Authors:** Shanting Hu, Hebin Zhang, Hui Ma, Cunxin Yang, Peipei Hu, Feng Gao

**Affiliations:** 1 Department of Ultrasonography, Affiliate Hospital of Hangzhou Normal University, Hangzhou, China; 2 School of Medicine, Hangzhou Normal University, Hangzhou, China; 3 Hangzhou Institute of Sports Medicine for Marathon, Hangzhou, China

**Keywords:** Marathon, Right ventricular function, Three-dimensional speckle tracking echocardiography

## Abstract

Prolonged high-intensity endurance exercise has been reported to have adverse effects on the heart, which are further correlated with exercise dose. However, its effect on the right ventricle (RV) of amateur runners is unknown. This study aimed was to evaluate the early right ventricular structure and systolic function of amateur marathon runners by three-dimensional speckle tracking echocardiography (3D-STE), and to further analyze the correlation between relevant parameters and the amount of training. A total of 30 amateur marathon runners (marathon group) and 27 healthy volunteers (control group) were enrolled. Conventional echocardiography combined with 3D-STE was performed in all subjects, and the marathon group was screened by echocardiography a week before a marathon (V1), within 1 h post-marathon (V2), and 4 days post-marathon (V3). RV global longitudinal strain (GLS) and RV end-diastolic volume (EDV) increased significantly in the marathon group compared to the control group (*P* < 0.05). RV GLS was significantly decreased in the marathon group within 1 h post-marathon (V1: − 26.2 ± 2.5% vs V2: − 23.0 ± 1.6% vs V3: − 25.6 ± 2.6%, *P* < 0.001). However, there was no significant difference in RV ejection fraction (RVEF) (*P* > 0.05). The results of the correlation analysis showed that RV EDV and RV end-systolic volume (ESV) were positively correlated with the average training volume (*P* < 0.001). Multivariate linear regression analysis showed that average training volume was an independent predictor of RV EDV in amateur marathoners (β = 0.642, *P* < 0.001). The systolic function of the RV was enhanced in amateur marathon runners in the early stage, manifested by an increase in RV EDV. After a long period of high-intensity endurance exercise, RV systolic function will temporarily be reduced. 3D-STE can identify this subclinical change with high sensitivity and provide valuable information to assess the structure and function of RV in amateur marathon runners.

## Introduction

A marathon is a popular long-distance exercise in China. With an increase in amateur marathon runners participating, there is growing concern about the effects of marathon running on the heart [1]. Regular and moderate exercise benefits physical health, while long-term high-intensity exercise may increase the risk of cardiovascular disease [2, 3]. Repeated high-intensity endurance exercise induces the body to produce a series of adaptation phenomena, such as enlargement of the heart cavity and thickening of the chamber wall, which is called the "athlete heart" [4]. The "athlete heart" has been reported to be a healthy and highly efficient adaptation to long-term high-intensity exercise [5]. However, left ventricular (LV) function has been confirmed to temporarily be impaired after intense exercise in professional athletes [6, 7]. Additionally, increasing evidence has shown that due to the different compensatory abilities of the pulmonary and systemic circulations, exercise-induced overload affects primarily the right ventricle (RV), and the effects are more apparent earlier than the effects on the left ventricle [8, 9], and some studies suggested the risk of "exercise-induced arrhythmia" [10–12].

Due to the irregular geometry and complex anatomical structure of the RV, it is challenging to quantitatively evaluate its function by two-dimensional echocardiography [13]. Three-dimensional speckle tracking echocardiography (3D-STE) is a new technology based on real-time three-dimensional echocardiography and two-dimensional speckle tracking technology, which can accurately and quantitatively assess myocardial motion and chamber capacity and detect subclinical changes in ventricular systolic function in the early stage with high sensitivity [14]. Previous studies have shown that the volume and strain values measured by 3D-STE are highly correlated with cardiac magnetic resonance [15].

Therefore, this study aims to analyze the early structural and functional changes of the right ventricular in amateur marathon runners by 3D-STE, to further elaborate the characteristics of heart remodeling in amateur marathon runners, and to provide a more in-depth theoretical basis for clinical guidance.

## Materials and methods

### Study population

From October 2020 to December 2022, we recruited 30 amateur marathon runners in Hangzhou, including 22 males and 8 females, aged between 27 and 51 years, with an average age of 38.77 ± 1.17 years. Clinical data such as age, body mass index, blood pressure, heart rate, competition times, and total training volume were recorded. The inclusion criteria are summarized as follows: (a) participating in marathon running for less than 3 years, (b) having completed a formal full marathon (42.195 km) at least once, (c) the average weekly running frequency ≥ 3 times, and the distance of each exercise ≥ 10 km, and (d) no strenuous exercise in the week before the marathon. Marathon runners were excluded if they had a history of congenital heart disease, hypertension, valvular heart disease, diabetes, kidney disease, and other systemic diseases. Marathon runners who have been involved in strength sports for a long time or were professional athletes were also excluded. 27 age- and sex-matched healthy sedentary subjects, including 21 males and 6 females, between 25 and 48 years old, with an average age of 36.00 ± 1.19 years were enrolled as controls. Control subjects had normal physical examinations, electrocardiograms, and echocardiograms. The Ethics Committee of the Affiliated Hospital of Hangzhou Normal University (Ethical approval number 2020E2-hS-001) approved the study. All participants had signed an informed consent form.

### Conventional echocardiography

We used EPIQ 7C color Doppler echocardiography diagnostic instrument (Philips Medical Systems) equipped with an X5-1 probe (1 ~ 5MHZ). Echocardiography was performed at the following time points: a week before a marathon (V1), within 1 h post-marathon (V2), and 4 days post-marathon (V3).

Conventional echocardiography was performed according to the American Society of Echocardiography (ASE) recommended guidelines. Parameters of the right atrial diameter(RAD), RV area and LV volume were obtained, and the LV ejection fraction (LVEF) was obtained by the biplane Simpson method from the apical two- and four-chamber views. In the RV-focused apical four-chamber view, tricuspid annular plane systolic excursion (TAPSE-2D) was measured in M-mode, the systolic velocity of the lateral tricuspid annular peak (S’) and the RV myocardial performance index (MPI) was measured by pulsed wave tissue Doppler imaging, and RV fraction area change (FAC-2D) was calculated as (RV end-diastolic area—RV end-systolic area)/RV end-diastolic area × 100%.

### Three-dimensional speckle-tracking echocardiography

**Fig. 1:**
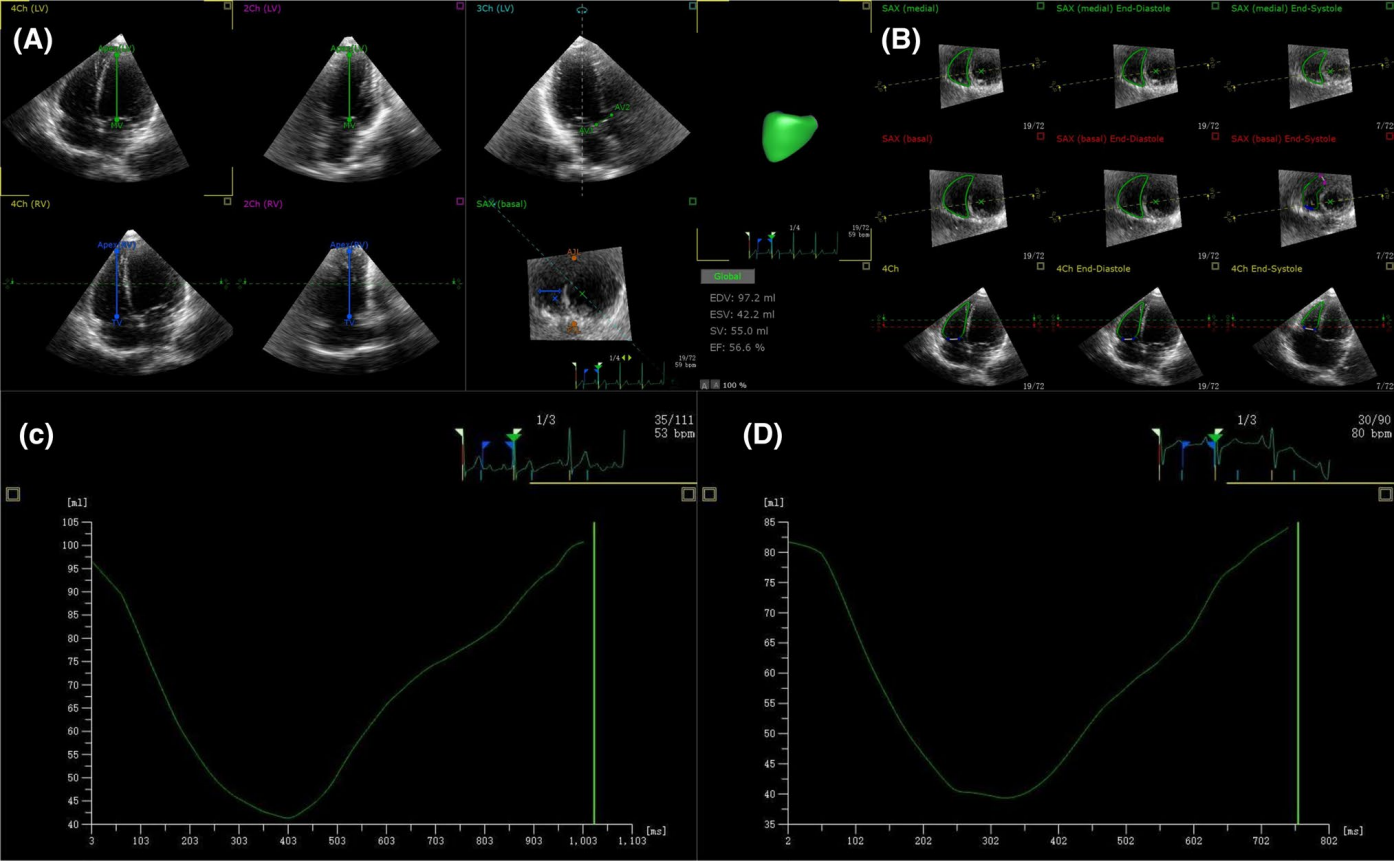
Three-dimensional speckle tracking imaging parameters of the right ventricle Offline analysis and volume-time curve. Manual determination of the points reference (A), track right ventricle endocardium (B), volume-time curve of the control group (C), volume-time curve of the marathon group (D)

Off-line analysis workstation TomTec 3.2 software (4D RV-Analysis 2.0, Germany). RV-focused 3D full-volume images were acquired with a frame rate > 60 frames/s. After manually determining the landmarks, the software automatically tracked the RV endocardium in the apical four-chamber and three short-axis views during the cardiac cycle, and then reconstructed the 3D model of the RV. If not satisfied with the tracking effect, you can adjust it manually. Finally, the software automatically generated RV end-diastolic volume (EDV), RV end-systolic volume (ESV), RVEF, TAPSE-3D, RV FAC-3D, RV free wall longitudinal strain (FLS), RV septal wall longitudinal strain (SLS), and obtained the RV volume-time curve (Fig. 1).

### Reproducibility analysis

Ten cases were randomly selected from the athlete group and the control group. The same observers measured the 3D-STE parameters two weeks later to evaluate the intraobserver differences. Differences between observers were evaluated by measuring relevant data by another experienced physician who was not involved in the study. The intraobserver and interobserver variations were expressed as correlation coefficients (ICC).

### Statistical analysis

Statistical analysis was performed using SPSS software version 22.0 (IBM, Chicago, IL, USA). The measured data was tested for normality using the Shapiro–Wilk test. Continuous variables were described as mean ± standard deviation for normally distributed variables and median and interquartile range (IQR) for nonnormally distributed variables. Categorical variables were described as frequencies and percentages and compared using Fisher’s exact test. For comparison between the two groups, the LSD-T test was used for variables that were normally distributed and the Mann–Whitney U test for variables with non-normal distribution. Differences between groups were compared using one-way ANOVA for continuous variables and the Kruskal–Wallis test for skewed variables. Spearman or Pearson’s correlation was used to test the correlation between clinical data and three-dimensional parameters. Multiple linear regression was used to analyze the predictive factors of RVEDV in amateur marathon runners. All statistical tests were two-sided and the *P* value < 0.05 was considered statistically significant.

## Result

### Clinical and demographic characteristics

A total of 30 amateur marathon runners and 27 healthy sedentary controls were included in the statistical analysis. Baseline demographic characteristics are summarized in Table 1. The heart rate of the marathon group was significantly lower than that of the control group (*P* < 0.05). There were no significant differences in age, body mass index, body surface area, systolic blood pressure, and diastolic blood pressure between the two groups (*P* > 0.05).

**Table 1:** Clinical and demographic characteristics of participants

Variable	Marathon group (*n* = 30)	Control group(*n* = 27)	*P* value
Age (years)	38.8 ± 6.5	36.0 ± 6.2	0.104
Male sex (%)	22 (73.3)	21 (77.8)	0.697
Body mass index (kg/m^2^)	22.6 ± 1.6	22.2 ± 1.1	0.182
Body surface area (m^2^)	1.7 ± 0.1	1.7 ± 0.1	0.640
Heart rate (bpm)	59.4 ± 3.9	76.9 ± 6.5	< 0.001
Systolic blood pressure (mm Hg)	114.8 ± 4.6	113.9 ± 4.1	0.474
Diastolic blood pressure (mm Hg)	75.4 ± 6.7	74.5 ± 6.3	0.706
Average training volume (km/week)	56.0 ± 6.9		
Average training time (h/week)	3.5 (3.0–4.0)		

Values are expressed as mean ± SD, N (%) or median (IQR)

### Conventional echocardiographic characteristic

The conventional echocardiographic parameters of the study population at baseline and during follow-up are shown in Table 2**.** The marathon group had significantly higher RAD1 and RAD2 compared to the control group (*P* < 0.05). However, there were no significant differences in LVEF, TAPSE-2D, S’ and RV FAC-2D (*P* < 0.05). Within the marathon group, TAPSE-2D was significantly lower and MPI was significantly higher within 1 h post-marathon compared to 1 week before a marathon and 4 days post-marathon (*P* < 0.05).

**Table 2:** Conventional echocardiographic parameters at baseline and during follow-up

	V1	V2	V3	Controls	*P*
RAD1 (mm)	5.0 ± 0.6*^,#^	4.6 ± 0.6	5.0 ± 0.7	4.4 ± 0.6	< 0.001
RAD2 (mm)	3.9 ± 0.7*^,#,&^	3.3 ± 0.5	3.6 ± 0.5	3.3 ± 0.7	< 0.001
LV EDV (ml)	137.2 ± 20.7	139.1 ± 16.1	137.5 ± 20.5	119.4 ± 7.3	0.170
LV ESV (ml)	47.5 ± 9.5	49.9 ± 4.5	47.5 ± 9.2	44.9 ± 4.6	0.659
LVEF (%)	65.4 ± 4.6	63.9 ± 2.5	65.5 ± 3.9	62.5 ± 2.0	0.313
TAPSE-2D (cm)	2.6 ± 0.2^#^	2.4 ± 0.1	2.5 ± 0.1	2.5 ± 0.1	0.015
S’ (cm/s)	16.0 ± 1.0	15.5 ± 0.9	15.9 ± 0.9	16.1 ± 0.7	0.083
RV FAC-2D (%)	39.9 ± 7.3	37.7 ± 8.1	40.3 ± 9.7	35.4 ± 15.7	0.820
MPI	0.45 (0.45–0.46) ^#^	0.53 (0.53–0.53)	0.46 (0.45–0.46)	0.45 (0.43–0.45)	< 0.001

Data are presented as mean ± SD or median (IQR)

V1 a week before a marathon; V2 within 1 h post-marathon; V3 4 days post-marathon

*RAD1* right atrial long diameter, *RAD2* right atrial transverse diameter, *LV EDV* left ventricular end-diastolic volume, *LV ESV* left ventricular end-systolic volume, *LVEF* left ventricular ejection fraction, *TAPSE-2D* tricuspid annular plane systolic excursion, *S’* the systolic velocity of the lateral tricuspid annular peak, *FAC-2D* fractional area change, *MPI* myocardial performance index

^*^p < 0 .05 compared to controls; ^#^p < 0.05 compared to V2; ^&^p < 0.05 compared to V3

### Three-dimensional speckle-tracking echocardiographic characteristic

**Fig. 2:**
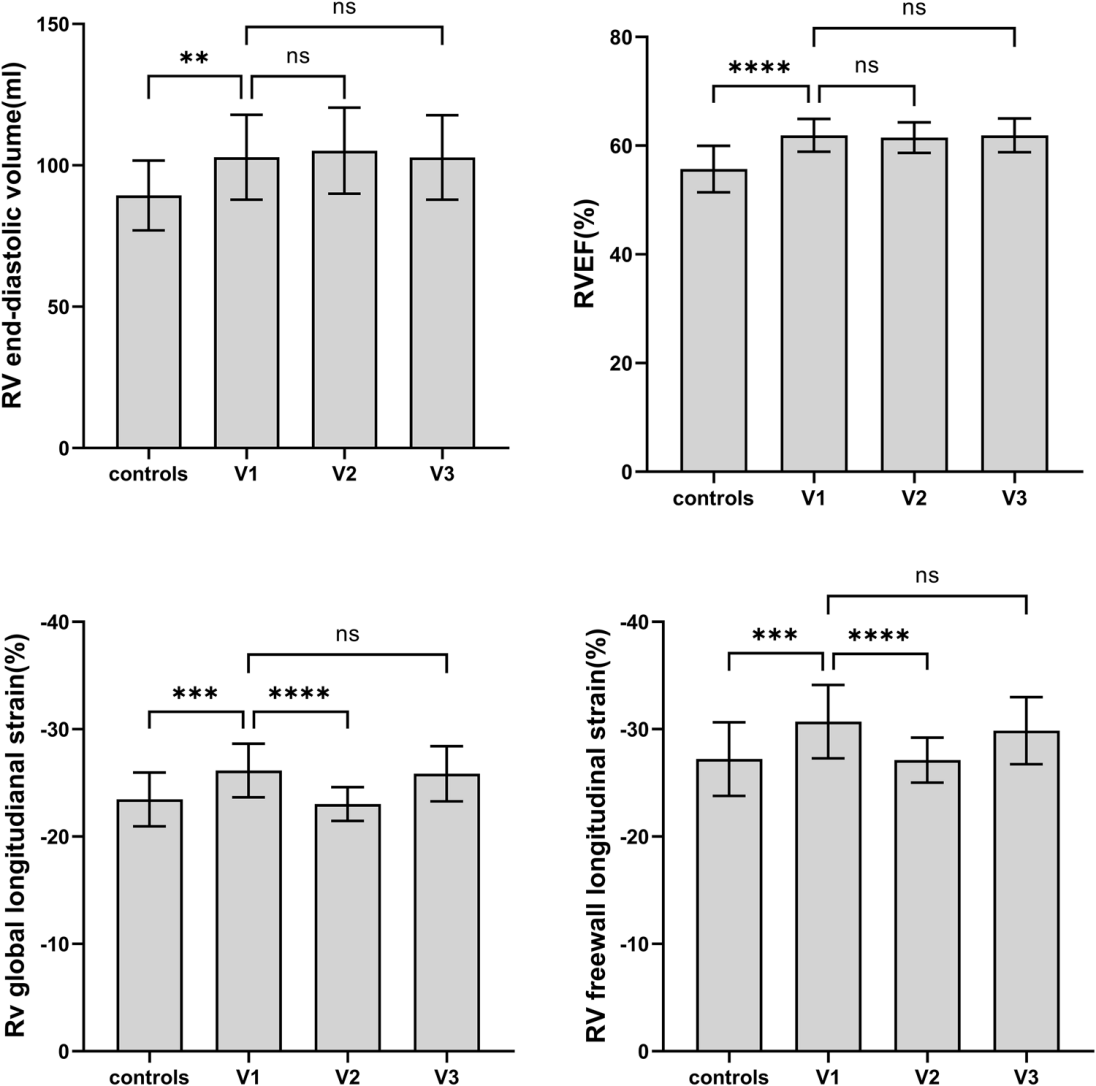
Comparison of the differences between the control group and the marathon group at baseline and during follow-up. For right ventricular end-diastolic volume, right ventricular ejection fraction, right ventricular global longitudinal strain, and right ventricular freewall longitudinal strain

The RV 3D-STE parameters are shown in Table 3 and Fig. 2. We found that RV EDV, RVEF, TAPSE-3D, RV FAC-3D, RV FLS, RV SLS and RV GLS of the marathon group increased significantly compared to the control group (*P* < 0.05). Within the marathon group, TAPSE-3D, RV FAC-3D, RV FLS, RV SLS and RV GLS within 1 h post-marathon were significantly lower than 1 week before the marathon and 4 days post-marathon (*P* < 0.05). However, there was no statistically significant difference in RVEF between the three time points of marathon runners (*P* > 0.05).

**Table 3:** Three-dimensional speckle-tracking echocardiography parameters

	V1	V2	V3	Controls	*P*
RV EDV (ml)	102.9 ± 15.0*	105.2 ± 15.2	102.8 ± 14.9	89.3 ± 12.3	< 0.001
RV ESV (ml)	39.3 ± 7.4	40.5 ± 6.6	39.2 ± 7.2	39.7 ± 7.1	0.897
SV (ml)	49.2 ± 3.0	49.6 ± 3.1	49.3 ± 4.8	48.8 ± 8.3	0.980
RVD1 (mm)	28.8 ± 1.6*	28.9 ± 1.9	28.7 ± 1.6	27.1 ± 1.9	< 0.001
RVD2 (mm)	31.1 ± 3.3	30.9 ± 3.3	31.1 ± 3.3	30.8 ± 3.3	0.975
RVD3 (mm)	75.99 (72.0–76.1)	75.5 (74.0–79.7)	75.3 (73.5–78.9)	75.9 (72.0–79.1)	0.825
RVEF (%)	61.9 ± 3.0*	61.5 ± 2.8	61.9 ± 3.1	55.7 ± 4.3	< 0.001
TAPSE-3D (mm)	24.9 ± 2.6*^,#^	23.1 ± 2.2	24.8 ± 2.4	22.8 ± 3.3	0.003
RV FAC-3D (%)	52.0 ± 3.1*^,#^	42.5 ± 3.9	50.6 ± 4.0	44.4 ± 6.2	< 0.001
RV FLS (%)	− 30.7 ± 3.4*^,#^	− 27.1 ± 2.1	− 29.9 ± 3.1	− 27.2 ± 3.4	< 0.001
RV SLS (%)	− 21.6 ± 2.5*^,#^	− 19.0 ± 3.2	− 21.8 ± 4.0	− 19.7 ± 2.6	< 0.001
RV GLS (%)	− 26.2 ± 2.5*^,#^	− 23.0 ± 1.6	− 25.6 ± 2.6	− 23.5 ± 2.5	< 0.001

*RV EDV* right ventricular end-diastolic volume, *RV ESV* right ventricular end-systolic volume, *SV* stroke volume, *RVD1* right ventricular basal segment diameter, *RVD2* right ventricular middle segment diameter, *RVD3* right ventricular long diameter, *RVEF* right ventricular ejection fraction, *TAPSE-3D* tricuspid annular plane systolic excursion, *RV FAC-3D* right ventricular fractional area change, *RV FLS* right ventricular freewall longitudinal strain, *RV SLS* right ventricular septal wall longitudinal strain, *RV GLS* right ventricular global longitudinal strain

^*^p < 0 .05 compared to controls; ^#^p < 0.05 compared to V2; ^&^p < 0.05 compared to V3

### Correlation analysis of amateur marathon runners

**Fig. 3:**
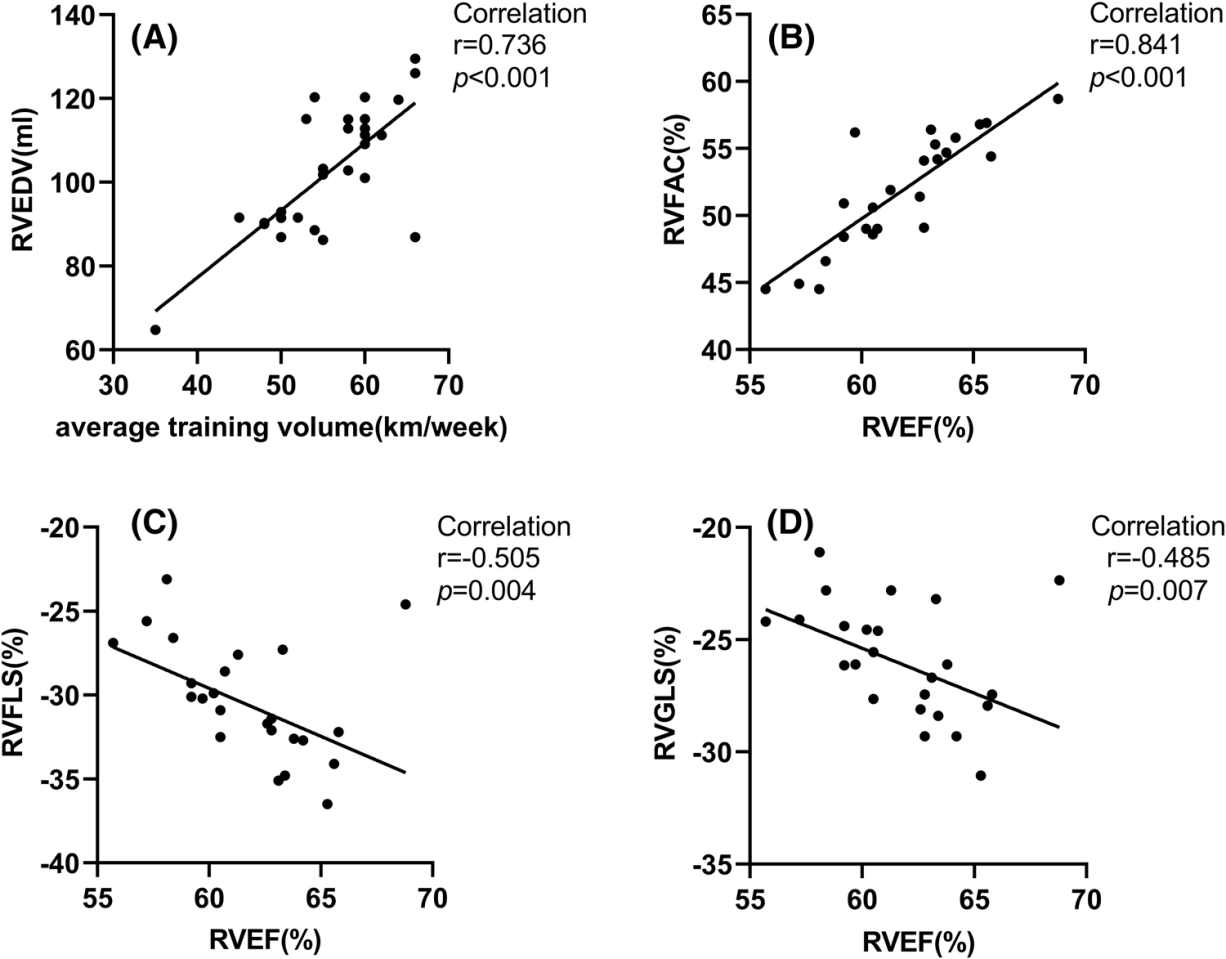
Correlation of RVEF and the average training volume with the 3D-STE parameters.Associations between RV EDV and the average training volume (**A**). Associations between RVEF and RV FAC (**B**); RV FLS (**C**); and RV GLS (**D**). *RV EDV*, right ventricular end-diastolic volume; *RVEF*, right ventricular ejection fraction; *RV FAC*, right ventricular fractional area change; *RV FLS*, right ventricular septal wall longitudinal strain; *RV GLS*, right ventricular global longitudinal strain

We found that RVEF and the average training volume were significantly correlated with the 3D-STE parameters in amateur marathon runners, the results of the correlation analysis are shown in Table 4 and Fig. 3. RV ESV (*r* = − 0.670, *P* < 0.001), RV GLS (*r* = − 0.485, *P* = 0.007) and RV FLS (*r* = − 0.505, *P* = 0.004) were negatively correlated with RVEF, while RV FAC-3D (*r* = 0.841, *P* < 0.001) was positively correlated with RVEF. RV EDV (*r* = 0.736, *P* < 0.001) and RV ESV (*r* = 0.638, *P* < 0.001) were positively correlated with the average training volume. Multiple linear regression analysis showed that the average training volume was an independent predictor of RV EDV in amateur marathon runners (β = 0.642, *P* < 0.001), as shown in Table 5.

**Table 4:** Correlation analysis of 3D-STE parameters with RVEF and average training volume of amateur marathon runners

	RVEF		Average training volume	
	*r*	*P* value	*r*	*P* value
RV EDV (ml)	− 0.306	0.100	0.736	< 0.001
RV ESV (ml)	− 0.670	< 0.001	0.638	< 0.001
TAPSE-3D (mm)	0.212	0.260	− 0.135	0.478
RV FAC-3D (%)	0.841	< 0.001	− 0.107	0.574
RV GLS (%)	− 0.485	0.007	0.146	0.440
RV FLS (%)	− 0.505	0.004	0.228	0.225
RV SLS (%)	− 0.273	0.144	− 0.019	0.919

**Table 5:** A multivariate regression analysis was performed to find predictive factors of RV EDV

	RV EDV	
	β	*P* value
Run of age (years)	− 0.126	0.421
Average training volume (km/week)	0.642	< 0.001
Average training time (h/week)	− 0.147	0.317
HR (bpm)	− 0.151	0.263
SBP (mmHg)	− 0.093	0.548
DBP (mmHg)	− 0.199	0.261
TAPSE-3D (mm)	0.145	0363
RV FLS (%)	0.187	0.291
RV SLS (%)	0.082	0.616

*HR* heart rate, *SBP* systolic blood pressure, *DBP* diastolic blood pressure, *RV EDV* right ventricular end-diastolic volume

### Reproducibility test

**Fig. 4:**
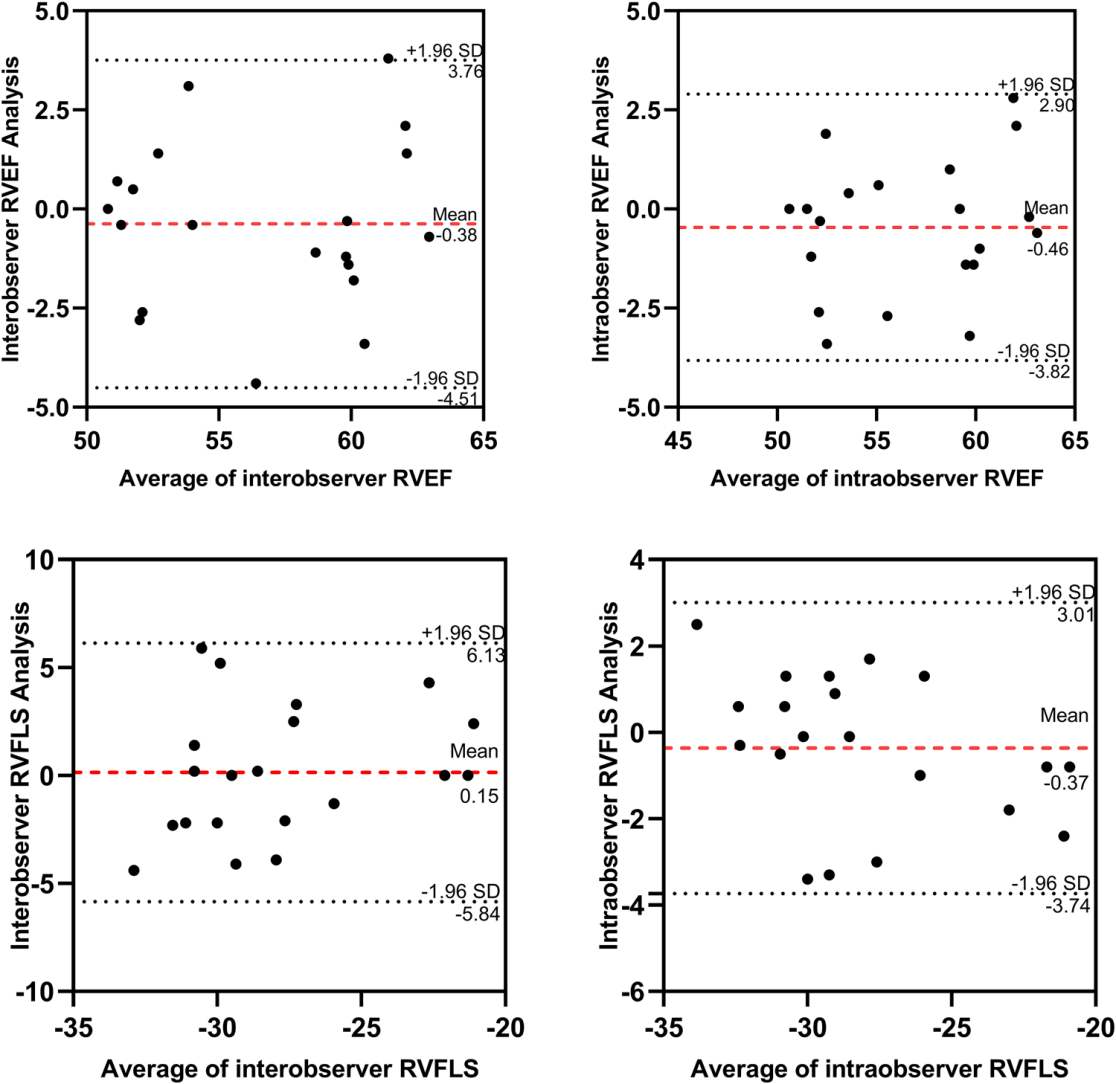
Bland–Altman analysis of RVEF and RV FLS. *RVEF*, right ventricular ejection fraction; *RV FLS*, right ventricular freewall longitudinal strain

The RV 3D-STE parameters were highly reproducible at both the interobserver and intraobserver levels. Interobservation measurements showed an interclass correlation of 0.82 for RV EDV, 0.89 for RVEF, 0.82 for RV FLS and 0.80 for RV SLS. Similarly, intraobservation measurements showed an intraclass correlation of 0.81 for RV EDV, 0.93 for RVEF, 0.91 for RV FLS and 0.84 for RV SLS, as shown in Fig. 4.

## Discussion

In this study, we performed a comprehensive and quantitative assessment of RV structure and systolic function in early amateur marathoners using 3D-STE. The main findings are as follows: (i). Amateur marathon runners have a higher early RV volume and better systolic function than the sedentary control group; (ii). The RV systolic function of amateur marathon runners may decrease transiently within 1 h post-marathon and would return to the baseline level 4 days post-marathon. 3D-STE is able to accurately identify this subtle change; (iii).RV volume was significantly associated with mean training volume in early amateur marathon runners, which has rarely been mentioned in previous studies.

We observed that the heart rate of amateur marathon runners was significantly lower than that of sedentary control. This lower heart rate in the resting state is likely due to long-term high-intensity endurance exercise increasing the vagal nerve tone of marathon runners and decreasing their sympathetic nerve tone [16], suggesting good cardiac reserve in the non-exercise state [17]. High-intensity endurance exercise will cause volume overload and increased atrial pressure [18]. In this study, the 3D-STE results showed that RV EDV, RVEF, TAPSE-3D, RV FAC-3D, RV FLS, RV SLS, and RV GLS of amateur marathon runners were higher than those of the control group. This result indicates that, to meet the body's demand for hyper ejection, the RV cavity is enlarged and the RV systolic function is enhanced in early amateur marathon runners [19]. Vitarelli et al*.* found [20] that patients with atrial septal defect also had enhanced right ventricular systolic function caused by chronic RV volume overload, and Esposito R et al*.* found [21] that the association between the longitudinal deformation of the free ventricle wall and the preload of rowers was independent of the effect induced by changes in afterload. These previous studies indicated that when the RV preload increased, the wall stress is maintained by the expansion of the chamber. Furthermore, the end-systolic volume is maintained at an early stage, while systolic function is enhanced through the Frank-starling law, which effectively compensates for changes in hemodynamic conditions [19, 22]. Studies have shown that the atrial pressure of professional athletes will be 7 ml/m^2^ higher than that of the general population [23]. Lewicka-Potocka et al*.* also found [4] that the left and right atrial sizes of recreational athletes were larger than those of a sedentary control group and were associated with the distance to training. In the present study, RAD1 and RAD2 also increased in amateur marathon runners compared to the control group, indicating that the size of RA would be compensated for with increased RV preload to allow greater volume delivery and increased cardiac output [24].

Our results showed that TAPSE-3D, RV FAC-3D, RV FLS, RV SLS, and RV GLS of amateur marathon runners were significantly lower within 1 h post-marathon, suggesting that under different load conditions, different changes in RV systolic function would lead to. During high-intensity endurance exercise, cardiac output is 7 times higher than in a normal resting state, which will inevitably lead to an increase in pulmonary vascular pressure and an increase in RV wall stress [9, 11]. Additionally, increased oxidative stress and myocardial damage together will cause transient RV systolic dysfunction [25]. However, there was no significant decrease in RVEF, which may be due to the compensatory increase in RV myocardium in other motor components, which allows RVEF to be within the normal range [26]. It is also further confirmed that 3D-STE can more sensitively recognize subtle damages to the RV myocardium.

In this study, we found that the 3D-STE parameters RV FLS, RV GLS, and RV FAC-3D of amateur marathon runners were significantly correlated with RVEF. Therefore, 3D-STE is a more accurate and reliable technique. We also found that RV EDV and RV ESV of amateur marathon runners were significantly correlated with the average training volume, suggesting that the early RV volume and RV systolic function of amateur marathon runners increased with the average training volume to meet the phenomenon of ventricular adaptation induced by endurance exercise [27]. This result fully reflects the intrinsic physiological characteristics of RV remodeling in amateur marathon runners [28]. The results of multivariate linear regression showed that the average training volume was an independent factor associated with the increase in RV EDV in amateur marathon runners. Recent data indicate that RV systolic function is impaired under high training load, suggesting the non-linear relationship between training volume and the incidence of cardiovascular complications [26]. We speculate that RV systolic function in the early stages may be manifested primarily by an increase in EDV, and when EDV reaches a certain threshold, RV systolic dysfunction occurs due to excessive volume, increased wall stress, and myocardial fibrosis [29]. Therefore, with the change in training volume in the early stage of amateur marathon runners, the RV of the body will produce adaptive remodeling but not intrinsic dysfunction.

## Limitations

This study only assessed amateur marathon runners in the early stages and the number of cases was limited, and professional marathon runners were not included for comparison. The index of cardiopulmonary function, such as maximum oxygen consumption (VO_2_ max), was not obtained. RV afterload parameters such as pulmonary artery pressure were not analyzed due to small tricuspid regurgitation. The statistical analysis of the parameters of each segment of 3D-STE longitudinal strain in amateur marathon runners has not been carried out, which will be further studied in the follow-up study.

## Conclusions

The longitudinal systolic function of RV of amateur marathon runners increases in the early stage, which is a good adaptation to endurance exercise. There are transient subclinical changes but no dysfunction in RV systolic function after high-intensity endurance exercise. 3D-STE is a practical and feasible echocardiographic technique to evaluate the dynamics of myocardial deformation, which is capable of identifying subtle changes in the myocardium comprehensively.

## Data Availability

The data in the current study are available from the corresponding author upon reasonable request.
